# Supplementary material for: Detecting variations in ovulation and menstruation during the COVID-19 pandemic, using real-world mobile app data
Source: PLoS One. 2021 Oct 20;16(10):e0258314. doi: 10.1371/journal.pone.0258314 (PMC8528316; doi:10.1371/journal.pone.0258314)
Supplement: Attachment — Submitted filename: COVID Cycles 2021_06_09 - PLOSONE Review Response.docx [file pone.0258314.s001.docx]

Dear editors and reviewers,

Thank you for your careful review of our manuscript, “Detecting variations in ovulation and menstruation during the COVID-19 pandemic, using real-world mobile app data.” We acknowledge the reviewers’ comments and have made revisions as appropriate with responses below. Please find our review responses in red.

-----------------------------------------------------------------------------------------------------------------------------

Dear Dr. Nguyen,

Thank you for submitting your manuscript to PLOS ONE. After careful consideration, we feel that it has merit but does not fully meet PLOS ONE’s publication criteria as it currently stands. Therefore, we invite you to submit a revised version of the manuscript that addresses the points raised during the review process.

The manuscript presents some interesting data. Before re-evaluating the possibility of publication, it is necessary that the authors provide feedback to the comments of the reviewers.

I also ask the authors to provide more details on the method the app uses to determine ovulation.

- We noted that other reviewers also requested more information on how the app determines ovulation and have included this clarification in more detail in the reviewer response below.

There is a comment the authors made claiming that their method has a Pearl index of 6.9, which they claim is comparable to hormonal contraceptives. This statement seems to be an advertisement for their app, and, as it is not strictly relevant to the content of the article, I think that has to be removed from the paper.

- Thank you for identifying this potential risk of bias. We removed the comparison to combined hormonal contraceptives. However, we kept the Pearl Index measurement, which is an objective measure.

------------------------------------------------------------------------------------------------------------------------------

**Journal Requirements:**

When submitting your revision, we need you to address these additional requirements.

1. Please ensure that your manuscript meets PLOS ONE's style requirements, including those for file naming. The PLOS ONE style templates can be found at

<https://journals.plos.org/plosone/s/file?id=wjVg/PLOSOne_formatting_sample_main_body.pdf> and

<https://journals.plos.org/plosone/s/file?id=ba62/PLOSOne_formatting_sample_title_authors_affiliations.pdf>

- Thank you for these links to the requirements. We have revised accordingly.

2. Please include your tables as part of your main manuscript and remove the individual files. Please note that supplementary tables (should remain/ be uploaded) as separate "supporting information" files

- We have incorporated the tables back into the manuscript. We have no supplementary tables, but will be attaching our data as supporting information

3.We note that you have indicated that data from this study are available upon request. PLOS only allows data to be available upon request if there are legal or ethical restrictions on sharing data publicly. For information on unacceptable data access restrictions, please see <http://journals.plos.org/plosone/s/data-availability#loc-unacceptable-data-access-restrictions>.

If there are no restrictions, please upload the minimal anonymized data set necessary to replicate your study findings as either Supporting Information files. Please see <http://www.bmj.com/content/340/bmj.c181.long> for guidelines on how to de-identify and prepare clinical data for publication. We will update your Data Availability statement on your behalf to reflect the information you provide.

- We will be including a minimal anonymized dataset.

4.Thank you for stating the following in the Financial Disclosure section:

"The authors received no specific funding for this work."

We note that one or more of the authors are employed by a commercial company: Natural Cycles

(a) Please provide an amended Funding Statement declaring this commercial affiliation, as well as a statement regarding the Role of Funders in your study. If the funding organization did not play a role in the study design, data collection and analysis, decision to publish, or preparation of the manuscript and only provided financial support in the form of authors' salaries and/or research materials, please review your statements relating to the author contributions, and ensure you have specifically and accurately indicated the role(s) that these authors had in your study.

You can update author roles in the Author Contributions section of the online submission form. Please also include the following statement within your amended Funding Statement. “The funder provided support in the form of salaries for authors [insert relevant initials], but did not have any additional role in the study design, data collection and analysis, decision to publish, or preparation of the manuscript. The specific roles of these authors are articulated in the ‘author contributions’ section.”

- We have revised our funding statement as follows:

“This is an investigator-initiated study by BTN, HRR, and ALN who have no commercial affiliation with Natural Cycles. While JA is an advisor for Natural Cycles, he has not received any salary or financial support for participation in this research. Natural Cycles provided support in the form of salaries for authors JTP, EBN, and AKvS, but did not have any additional role in the study design, data collection and analysis, decision to publish, or preparation of the manuscript. The specific roles of these authors are articulated in the “author contributions” section.”

(b) Please also provide an updated Competing Interests Statement declaring this commercial affiliation along with any other relevant declarations relating to employment, consultancy, patents, products in development, or marketed products, etc.
    Within your Competing Interests Statement, please confirm that this commercial affiliation does not alter your adherence to all PLOS ONE policies on sharing data and materials by including the following statement: "This does not alter our adherence to PLOS ONE policies on sharing data and materials.” (as detailed online in our guide for authors http://journals.plos.org/plosone/s/competing-interests) .
    If this adherence statement is not accurate and there are restrictions on sharing of data and/or materials, please state these. Please note that we cannot proceed with consideration of your article until this information has been declared.

- Statement included accordingly

(c) Please include both an updated Funding Statement and Competing Interests Statement in your cover letter. We will change the online submission form on your behalf.

- Statement included accordingly

5. Please amend either the abstract on the online submission form (via Edit Submission) or the abstract in the manuscript so that they are identical.

- We have shortened the abstract in the manuscript to 300 words and will submit accordingly.

**Review Comments to the Author**

*Reviewer #1:* 
The authors present a very large data set, collected through the app Natural Cycles, which provides a firm basis for real world conclusions. The data herein details changes in menstrual functionality amongst women during the COVID pandemic, comparing to data pre pandemic. The aim and relevance of the data collected is clear and necessary, however there are queries amongst how the data is collected through the app to determine ovulation. This needs to be clarified in more detail within the manuscript. Please see specific comments below.

Abstract:

The first sentence of the abstract needs to be reworded or include more commas, as it stands this is not a very clear start to the manuscript.

- We have adjusted the abstract completely in order to better fit the word limit for PLOS ONE. The first sentence now reads:
  “As war and famine are population level stressors that have been historically linked to menstrual cycle abnormalities, we hypothesized that the COVID-19 pandemic could similarly affect ovulation and menstruation among women.”

The authors state they restricted the data set to regular cycling women yet do not include the clear, readily used, definition of eumenorrheic. The authors should detail which definition they have used in the abstract. Further comments on this below.

- Given the word limit, we could not go into detail about this in the abstract, though have revised the manuscript to provide more detail as requested. Please see more details below.

Ethics statement: The authors have stated that it was clarified that this was a human exempt study, could the authors make it explicitly clear that whilst this study was exempt that ethical approval was still sought?

- We updated and clarified the ethics statement as follows:
  “This research plan was reviewed and approved by the USC Institutional Review Board, which classified the study as exempt, non-human subjects research (HS-20-00402).”

Introduction
Line 123: ‘The events set…’ this depicts that it was the lockdowns that caused the concerns about the pandemic alone, in fact there were many different situations and events on going across the globe and collectively that raised the levels of concern. I would suggest re structure to ensure this comes through in the text.

- We changed the verbiage from “set off” to “contributed to” in order to mitigate suggestions of complete causality:
  “These events contributed to population level concerns about the risk of not only infection and loss of life, but the potential irrecoverable loss of livelihood, as non-essential businesses were shut down and citizens deprived of income, while facing continued expenses.”

Line 128: …’ability to reproduce’ I can see the authors need to introduce this concept to the text at this point as to the purpose of their manuscript however the placement of this here does not fit. The ability to reproduce was certainly not an immediate outcome of the pandemic nor do we yet have the scientific data to suggest this is a consequence of the long COVID for example. I would suggest either removing or expanding here on how the pandemic directly affects the ability of people to reproduce.

- We have removed this statement.

Line 139: …’transmitted generationally’ please provide a reference here that demonstrates the affects of stress on the menstrual cycle can be transmitted between generations.

- Thank you for catching this. We wanted to express that events occurring in utero and in childhood could manifest as menstrual changes in later life. Though given that this was not the focus of this study, we removed this statement.

Line 147: typo.

- Changed from “patters” to “patterns”

Line 157-161. The last sentence of this section needs to be re worded, it is not coherent in its current format. This sentence is making quite assumptive statements too I would suggest being more direct in making your point and include references.

- We removed the statement about associations with heavy menstrual bleeding and impaired fertility. These are potential clinical implications of menstrual changes that we suggested early in order to bring clinical relevance to this study, which may not be appropriate at this juncture of the paper.

Line 177: Reasonably certain? Can the authors provide clarification or quantification to this?

- The Pearl Index is the quantification of contraceptive efficacy that provides reasonable certainty that this method is effective.

Methods:
The main issue here is the lack of clarity on determining ovulation. This is one of the main aims/outcomes of the study yet there are no specific details on how ovulation was detected through the app. There are some suggestions of basal body temperature, but no detail. This is imperative to be provided. Were all users testing temperature at same time of day? Were they using the same device? Did they all test the temperature regularly? If not how often? Without such information it makes the data seem quite questionable as to how the results can be deemed valid and/or appropriate.

- We have provided more detail about how basal body temperatures are recorded as follows:

“Anovulatory cycles were defined according to the Natural Cycles app’s proprietary algorithm. The algorithm identifies ovulation retrospectively based on the first day of menstruation and basal body temperatures, which may be supplemented by positive urinary LH tests. Basal body temperatures are recorded each morning using a thermometer sensitive to the hundredth place, and with measures excluded if the user reports any illness or changes in sleep that might influence basal temperatures. Users of Natural Cycles record basal temperatures for approximately 70% of the days. To reduce the risk of misidentifying ovulations, the algorithm reports ovulation by rising basal body temperature only if the average temperature from three consecutive calendar days is greater than the woman’s follicular phase average and her baseline average across all data entries, as well as consistent with her luteal phase average [15]. If no temperature rise is observed and the data quality and quantity is deemed sufficient, the cycle is flagged as anovulatory. Cycles with low data quality or many missing data points are excluded by the analysis.”

Line 239. The definition of normal cycles often used in research is 21-35 days long, please could the authors explain why they chose 24 days as the shortest cycle to include?

- We used criteria from FIGO (<https://pubmed.ncbi.nlm.nih.gov/17362717/>), which are based on more recent population estimates at the 5^th^ and 95^th^ percentile. Had we used 21 days, we would have significantly underestimated frequent menstruations and potentially been less likely to find a difference.

Line 254-255: The covariates stated are all reasonable but again no information of the questions as to how this was collected or categorised. Please provide such detail.

- We respectfully disagree w/ the reviewer about the lack of details on the covariates. We provide the exact questions asked regarding our primary covariate of interest on pandemic-related stress. We discuss how users are asked sociodemographic items at the time of registration and how these data are then used for our analysis. With concision in mind, we do not believe that providing verbatim questions about how socio-demographics are collected is relevant to the reader’s interpretation of the validity of these items. However, we did provide categories for some of the covariates as follows:

“In addition, we used sociodemographic data and reproductive histories provided by users at the time of app registration and updated during use of the app as covariates in our analysis. These covariates included: age, country of registration, education (from less than a high school degree to graduate degree), relationship status (e.g., in a relationship, engaged or married, it’s complicated, and single), history of pregnancy, and whether they have any children.”

Results
The display of data is very poor.
Figure 1 in particular has different coloured text/font/size. The key does also not correspond with the data being shown. Please revise thoroughly.

- We revised the figure for consistent font and font size throughout. The reviewer however may not fully understand how to interpret the key, whereby “More” and “Fewer” refers to the proportion of individuals who had more versus fewer abnormal cycle parameters reported. The goal of the figure is to show that users more frequently reported fewer abnormal parameters. We expanded the key to read “More abnormal parameters” and “Fewer abnormal parameters” to assist with the confusion.

The symbols used in the tables to display significance, it is not clear which category this is referring to compared to which other category. Please revise.

- We believe that the reviewer is referring to Table 1 where we indicate significance at the level of the variable itself across all categories. The reviewer may be confused about the test performed in this table. We conducted Chi-square tests here where the comparison does not provide measure of significance by each category, rather the significance refers to the relationship between the two variables.

Table IV is also quite confusing as to whether this is referring to categories of stress or not. I would suggest changing to a clearer display method or altering the table/better legends.

- The reviewer is asking for a simpler table and perhaps one that only focuses on stress. I disagree with the reviewers’ suggestion as it is important for the reader to see all covariates that were included in the regression modeling, particularly because stress was not ultimately a statistically significant contributor to abnormal cycle parameters while others, such as relationship status and age were. We recognize that the majority of Table IV does not provide significant findings, however we believe that presenting the data in this way is important for transparency.
- We did however change the title to better explain the column headings to: “Factors linked to more versus fewer cycle abnormalities during the COVID-19 pandemic”

Line 293: include the significance value.

- We omitted the significance value purposefully because the magnitude of the p-value does not provide any information on strength of relationship and because we list multiple factors for which the p-values differ from p<0.05 to p<0.001. The reader who does not trust the text can see Table 1 for immediate clarity on the p value.

Lines 299-301 The authors state a significant change in cycle length yet looking at the numbers this changes by 0.5 day, the authors need to clarify the length of cycles users inputted as it seems somewhat strange that a user would have a cycle length of 29.5 days.

- The cycle lengths are averaged from the number of cycles that are contributed over the study periods, which is why the averages are fractions of whole numbers.

Line 301: Please include here the number of ‘average incidence’ of ovulation.

- We did not record ovulations specifically. Our methods were aimed at specifically recording anovulations and we have provided more detail on the changes as follows:
- “The average incidence of anovulation and abnormal cycle length decreased significantly across cycles, from 2.9% (2.7%-3.0%) to 2.5% (2.3%-2.6%) and 8.7% (8.4%-8.9%) to 8.0% (7.8%-8.2%), respectively, while the average incidence of prolonged menstruations increased from 0.9% (0.8%-1.0%) to 1.0% (9.0%-1.1%).”

Line 319: Indicates that more variable cycles came from older participants, yet the authors have not discussed later that this could be linked to peri menopause as well. I would suggest mentioning this as a possibility within the discussion.

- We have added a statement as follows to address the reviewer’s comment:
- Even when examining the cycle characteristics of women self-reporting more (32.0%) and markedly more (13.6%) stress following the pandemic in a multivariable logistic regression, we did not observe any association of stress with cycle abnormalities. Rather than among women reporting high stress, we noted more abnormal cycles among women greater than the age of 45 years during the pandemic, which may suggest the stress-related sensitivity of the hypothalamic-pituitary axis during the perimenopausal state.

Discussion:
As per above there needs to be more a more detailed discussion of the possible limitations which should include the ovulation reliability, possibility of peri menopause, as well as the possible bias to the data set as already included. I would suggest a specific limitation section should be included as the whole basis of the data collection is reliant upon people taking their own ovulation testing and truthfully inputting the data. There are many studies that have discussed the inaccuracies of self-report data but none have been acknowledged or references. Please include such.

- We note that the reviewer is very focused on a biased critique of the app being unable to accurately predict ovulation. We note that ovulation in all the possible ways in which it can be measured non-invasively are soft markers. Even the gold standard of using ultrasound cannot determine if a ruptured follicle has released an egg. To suggest that our research would be improved via a study that demanded transvaginal ultrasound and routine LH kit testing would be infeasible at our sample size. Ovulation prediction is limited, which is why our investigation additionally includes other menstrual parameters aimed at increasing our ability to detect changes related to the pandemic. The reviewer’s comments do not acknowledge that this data set is one of largest to collect these data and that any data collection performed in a clinical setting would be subject to even greater limitations. Any paper-collected self-report would be even less reliable and more likely to reflect recall bias, which our methods avoid by requiring daily data entry. Abiding by the reviewer’s request would turn a strength of our methodology into a weakness.
- That being said, we have added the following broad limitation acknowledgement in a separate limitations section as follows:

“The findings from this study are broadly limited by self-reported data. However, we believe that these data are collected from a group of individuals who are using the Natural Cycles app for its intended purpose of pregnancy prevention, rather than for the purposes of this research, such that these data are expected to be reliable.”

Line 368: In a May 2020…Please re word.

- We have revised as follows:
- “In a survey of approximately 1800 majority college-educated, employed US adults in May of 2020, more than one-third reported engaging in more physical activity and getting more sleep, which they attributed to having more time and less stress during the pandemic.”

A clearer conclusion/future work section needs to be included, the manuscript ends somewhat abruptly.

- We have included a conclusion section as follows:
  “The COVD-19 pandemic did not induce population-level changes to ovulation and menstruation among women using a mobile app to track menstrual cycles and predict ovulation. While some women experienced abnormalities during the pandemic, this proportion was smaller than that observed prior to the pandemic. As most app users in this study were well-educated women over the age of 30 years, and from high-income countries, their experience of the COVID-19 pandemic is expected to differ in ways that limit the generalizability of these findings.”

---------------------------------------------------------------------------------------------------------------------------------

Reviewer #2:

This paper is quite interesting, and addresses a very commonly asked question by women during the pandemic. The authors do discuss the limitations of their paper, as far as the study group (Highly educated, presumably well off, young women, in general) but they have a considerable data base. And their negative findings are very interesting. As an ancillary question, which has nothing to do with the current study: I would ask, perhaps from a sociologist or psychologist, why this group of women in 2019 (going back to their control group) were experiencing so much stress? If I read the numbers correctly, almost half (46.2%) of the women studied stated that they were either very or extremely stressed in 2019. Why? But I think the study as is quite succinct and helpful.

- We appreciate the reviewer’s interest in this study. While we cannot be sure, the anxiety felt by women from 2019 might reflect a selection bias whereby users consistently using the app and providing data across this entire time period might have greater baseline anxiety.

------------------------------------------------------------------------------------------------------------------------------------

Reviewer #3: This is a very important and time-sensitive topic that has received a lot of media attention for anecdotal report of menstrual changes during the COVID-19 pandemic. It is great to have data to start to answer some of these questions. This manuscript contains valuable information but needs editing and some clarifications to make it suitable for publication.

Overall comments:
1. The entire paper is too long and wordy. It would benefit from editing, especially in the Abstract, Introduction, and Discussion. More details below.

- We have markedly shortened our abstract and paid attention to removing elements of the introduction that might be too broad in their explanation of the background (e.g. US and UK stay at home orders during pandemic), or were outside the scope of our investigation (e.g. birthrates). We additionally shortened our discussion.

2. Better clarification is needed about the definition of anovulation. While it appears that you are using definitions of ovulation/anovulation as defined by a confidential algorithm, it is confusing because regular menses and cycle length are typically used as proxies for ovulatory cycles, and yet anovulation and abnormal cycle length and prolonged menses are presented as completely different concepts here.

- Natural Cycles is advantageous for its use of BBT and LH results as proxy for ovulation, which is a more direct way of assessing ovulation than regular menses and cycle length.

3. Similarly, you seem to imply that regular menstrual cycles was an inclusion criterion but then report anovulatory cycles in this cohort. I believe that you are excluding amenorrhea or “very irregular” cycles but need to clarify these concepts and definitions better.

- We clarify this in our methods as follows:
  “To account for any undiagnosed amenorrhea or ovulatory disorders, as well as to improve our ability to detect pandemic-induced changes in ovulation and menstruation, we excluded users who recorded any cycle length lasting more than 90 days during the pre-COVID analysis frame. We further excluded users who did not contribute at least two cycles of data prior to the COVID-19 pandemic and at least two cycles during the COVID-19 data collection period.”

Abstract:
-Much too long. The abstract should not be longer than 300 words and this seems much longer.
-Subheadings are typically Background, Methods, Results, Conclusions. There is no summary/conclusions in your abstract.
-Limitations are not included in the abstract.
-Have never seen an Extended Abstract before. Was this specifically requested? If not, please remove. Your abstract should speak for itself and lead readers to read the entire paper.

- We have revised the abstract completely to match the PLOS ONE guidelines. We appreciate the reviewer’s comments above and have edited accordingly though note the importance of including the limitation of selection bias in the abstract given that the readership may sometimes only read the abstract and major limitations should be included.

Introduction:
-Too long, should not be more than 2-3 paragraphs. Needs editing.

- We reviewed several manuscripts from PLOS ONE, noting that very few had introductions that were less than 4 paragraphs. We respectfully disagree w/ the reviewer given that we feel that our introduction is very informative of this phenomenon and its biological plausibility, which then give rationale for this study. However, we did find other ways to markedly shorten the introduction.

-Last paragraph should be moved to Methods.
-Section should end with a clear statement of the objective of this study.

- We understand that because this paragraph introduces the app and some of its characteristics that the reviewer might prefer to see this in the methods, however the app’s early introduction is essential to the study question and objective. We use the methods to discuss how the app works and the data it collects instead.
- However, we did move some of the clarifying information about the app’s contraceptive efficacy to the methods.

Methods
-The Inclusion/Exclusion criteria section is long-winded and confusing. Needs to be tightened up and clearer. The most confusing part is the exclusion of those with any cycle >90 days “to account for any undiagnosed amenorrhea or ovulatory dysfunction.” This needs to be clarified. I believe what you mean is that you excluded anyone with secondary amenorrhea, as defined by any cycle >90 days. You did not exclude anyone with irregular cycles, as those would have been anyone with cycles <24 or >38 days, per the FIGO definitions you cite on the next page. As such, the line in the Outcomes section about “… the proportion of users experiencing anovulation, abnormal cycle length, and prolonged menstruation among regularly cycling app users …” seems contradictory. The phrase “regularly cycling app users” should be eliminated throughout the manuscript, as you are not necessarily including those with regular cycles, just those who are not amenorrheic.

- We clarified that we are looking at a population of users without any history of amenorrhea. We removed “regularly,” as suggested by the reviewer and appreciate their attention to detail.

-Why were women with endometriosis excluded? What is the evidence for endometriosis affecting menstrual regularity?

- For this type of study, we want to keep our sample restricted to those who have high likelihood of regular ovulation. Individuals with endometriosis could have endometriomas that affect the ovary or could have pain conditions that warrant hormonal management. Endometriosis was excluded less for menstrual affects, but rather its potential ovulatory effect.

-I assume that women using this app are not on any hormonal birth control method, but that should be explicitly stated.

- Revised as requested

-The last 3 sentences of the Inclusion/Exclusion section are redundant and should be removed.

- There are very important details in each of these 3 sentences. Readers need to know why we selected our time frames as we did, omitting Oct 2019 through Feb 2020 because of the ambiguity of concerns about the pandemic during these months. Further, readers need to know that our determinations of ovulation are aimed at improving accuracy via only looking at validated cycles where sufficient BBT was reported.

-Outcome section: Please clarify the line “which may be supplemented by positive urinary LH tests.” Was this done for all women?

- The main inputs to our algorithm are period dates and BBT. However, users are encouraged to optionally take LH tests during the cycle and log the results in the app. If they do, the results are also used in the algorithm. Around 25% of our users use LH tests.

Results
-Be consistent in the text and tables about the numbering system for tables. You use Arabic numbers (e.g. 1, 2) in the text but Roman numerals (e.g. I, II) for the tables themselves.

- Revised to Arabic

-If you state that there is a “significant” difference in the text and include numbers/percentages for the groups, then you should also include p-values in the text. This is very inconsistent.

- We respectfully disagree w/ the reviewer’s blanket recommendation as we purposefully omitted some p-values where the findings were not CLINICALLY significant. Because we know that p values can be misleading w/ respect to their suggestion of differences, we were careful about where we mentioned p-values in the text, acknowledging as well that p-values may not always be an appropriate measure of magnitude of difference.

-There is no reason to include both Figure 1 and Table 3, as both give the same information. I think it is presented better in the figure.

- We respectfully disagree w/ the reviewer’s statement that there is no reason for both the Figure and Table. The Figure is helpful for its focus on users with more stress, to provide a visual understanding of there being comparable proportions of individuals with more and fewer abnormal cycle parameters before and during the pandemic. The Figure is important for the primary hypothesis. Table 3 is included to be thorough and incorporate data on individuals experiencing less stress or no change in stress. Should the editor prefer, this could be a supplementary table, but as is, we are not compelled to remove it.

Discussion
-This is too long. The purpose of the Discussion is to summarize and interpret your most significant findings and compare them to prior data. You should not rehash all the Results.
-You take almost 2 full pages at the end of the Discussion to make the same point. 1) This should be significantly shortened. 2) You must also consider the possibility that routine stress, as experienced by many during COVID-19, is different than the stress experienced during, for example, war and that COVID-19 might not have caused significant physiologic stress and might not have had any affect on menses and ovulation.

- We have significantly shortened the discussion per reviewer request.
- We have already addressed the reviewer’s comment about routine stress being insufficient in our manuscript as follows:
  “ For this population, general stress provoked by the COVID-19 pandemic and its preoccupations may not have been sufficient to elicit an adrenal and adaptive neuroendocrine response to acute stress or chronic stress”

-The final paragraph is out of place as the concluding paragraph. Shorten to 1-2 sentences as additional limitations and add a true Conclusions final paragraph to the paper that summarizes the importance of your findings and next steps.

- We have edited as requested and included a final Conclusions paragraph.

Tables
-Consistent numbering, per comment above

- Revised to Arabic

-Why does Table 2 have an * in 1 place? What does this mean? Needs to be defined under table.

- This was a typo that we removed. Thank you.
