# Supplementary material for: Detecting variations in ovulation and menstruation during the COVID-19 pandemic, using real-world mobile app data
Source: PLoS One. 2021 Oct 20;16(10):e0258314. doi: 10.1371/journal.pone.0258314 (PMC8528316; doi:10.1371/journal.pone.0258314)
Supplement: Attachment — Submitted filename: COVID Cycles 2021_09_01 - PLOSONE Review Response.docx [file pone.0258314.s002.docx]

**PLOS ONE Review Response #2**

Dear editors and reviewers,

Thank you for your careful review of our manuscript, “Detecting variations in ovulation and menstruation during the COVID-19 pandemic, using real-world mobile app data.” We acknowledge the reviewers’ comments and have made revisions as appropriate with responses below. Please find our review responses highlighted in yellow.

1. If the authors have adequately addressed your comments raised in a previous round of review and you feel that this manuscript is now acceptable for publication, you may indicate that here to bypass the “Comments to the Author” section, enter your conflict of interest statement in the “Confidential to Editor” section, and submit your "Accept" recommendation.

Reviewer #1: (No Response)

Reviewer #3: (No Response)

2. Is the manuscript technically sound, and do the data support the conclusions?

The manuscript must describe a technically sound piece of scientific research with data that supports the conclusions. Experiments must have been conducted rigorously, with appropriate controls, replication, and sample sizes. The conclusions must be drawn appropriately based on the data presented.

Reviewer #1: Yes

Reviewer #3: Yes

3. Has the statistical analysis been performed appropriately and rigorously?

Reviewer #1: Yes

Reviewer #3: No

- Please see responses to reviewer below to address concerns about statistical analysis/methods

4. Have the authors made all data underlying the findings in their manuscript fully available?

The [PLOS Data policy](https://urldefense.com/v3/__http:/www.plosone.org/static/policies.action*sharing__;Iw!!LIr3w8kk_Xxm!-_bfMTl4-Gu0gC0t2RJ6KbytuyVi-qFyn179-vykm_0zm7tjUKe8VXoFIwTVza0$) requires authors to make all data underlying the findings described in their manuscript fully available without restriction, with rare exception (please refer to the Data Availability Statement in the manuscript PDF file). The data should be provided as part of the manuscript or its supporting information, or deposited to a public repository. For example, in addition to summary statistics, the data points behind means, medians and variance measures should be available. If there are restrictions on publicly sharing data—e.g. participant privacy or use of data from a third party—those must be specified.

Reviewer #1: Yes

Reviewer #3: Yes

5. Is the manuscript presented in an intelligible fashion and written in standard English?

PLOS ONE does not copyedit accepted manuscripts, so the language in submitted articles must be clear, correct, and unambiguous. Any typographical or grammatical errors should be corrected at revision, so please note any specific errors here.

Reviewer #1: Yes

Reviewer #3: Yes

6. Review Comments to the Author

Please use the space provided to explain your answers to the questions above. You may also include additional comments for the author, including concerns about dual publication, research ethics, or publication ethics. (Please upload your review as an attachment if it exceeds 20,000 characters)

**Reviewer #1: Thank you to the authors for address the majority of the queries put forward by both reviewers. The manuscript provides a lot more detail in its current format. I do still have some concerns/queries that need to be addressed which I have provided in the comments below (see marked comment 2).**

Original comments: Methods: The main issue here is the lack of clarity on determining ovulation. This is one of the main aims/outcomes of the study yet there are no specific details on how ovulation was detected through the app. There are some suggestions of basal body temperature, but no detail. This is imperative to be provided. Were all users testing temperature at same time of day? Were they using the same device? Did they all test the temperature regularly? If not how often? Without such information it makes the data seem quite questionable as to how the results can be deemed valid and/or appropriate.

Original response: We have provided more detail about how basal body temperatures are recorded as follows: “Anovulatory cycles were defined according to the Natural Cycles app’s proprietary algorithm. The algorithm identifies ovulation retrospectively based on the first day of menstruation and basal body temperatures, which may be supplemented by positive urinary LH tests. Basal body temperatures are recorded each morning using a thermometer sensitive to the hundredth place, and with measures excluded if the user reports any illness or changes in sleep that might influence basal temperatures. Users of Natural Cycles record basal temperatures for approximately 70% of the days. To reduce the risk of misidentifying ovulations, the algorithm reports ovulation by rising basal body temperature only if the average temperature from three consecutive calendar days is greater than the woman’s follicular phase average and her baseline average across all data entries, as well as consistent with her luteal phase average [15]. If no temperature rise is observed and the data quality and quantity is deemed sufficient, the cycle is flagged as anovulatory. Cycles with low data quality or many missing data points are excluded by the analysis.”

Comment 2: Thank you for providing this important detail. Could the authors please clarify the distribution of reporting further. The authors state that basal temperatures are recorded approximately 70% of the days, it is imperative to provide detail on this, what days were reported more than others? Did it vary between participants using the app? If certain days were continually omitted in some cases then this would contribute somewhat to the outcomes of the analysis.

Users are instructed to avoid measuring and including their temperature if they have been drinking or if their sleeping pattern varies. Both scenarios are more likely to happen during the weekend, when users tend to go out more often. We therefore see a decrease of temperature data logged on Saturdays and Sundays. However, we do not believe that this will affect the outcome of the analysis, as we are looking at the fraction of anovulatory cycles. Let’s consider for example a user who didn’t log any temperature over the weekend, and has ovulated on Sunday. Due to the missing data we will not know exactly on which day the user ovulated, but we can still detect that ovulation has happened by looking at the elevated temperatures following ovulation. Being able to determine if ovulation has happened is the only relevant information when computing the fraction of anovulatory cycles.

We specify this lack of impact on the overall reliability of our primary outcome as follows:

“If no temperature rise is observed and the data quality and quantity is deemed sufficient, the cycle is flagged as anovulatory. Cycles with low data quality (e.g., high day-to-day temperature variability) or many missing data points are excluded by the analysis. *While missing data may affect the app’s ability to predict the exact day of ovulation, they are not expected to affect its determination of whether ovulation has occurred.* Based on criteria from the International Federation of Gynecology and Obstetrics (FIGO), we defined abnormal cycle length as lasting less than 24 days or more than 38 days, and prolonged menstruation length as lasting greater than 8 days [12][13].”

Comment 2: The authors also state that cycles will low data quality or any missing data points are excluded by the analysis. Again the detail is required here. What was the authors definition of low quality data (is this linked to the statement prior to this or something else?) and provide exactly how many missing data points that then meant the cycle was excluded.

In order to either detect ovulation or flag a cycle as anovulatory, we require a minimum amount of good quality temperature measurements. In fact, our ability to detect a statistically significant increase of the BBT in the luteal phase is affected by both the data quantity and quality, and the adopted criteria depends on a complex interplay of these two factors. If the user usually has stable measurements (with small day-to-day variations in the same cycle phase), the algorithm will be able to draw conclusions with fewer data points. On the contrary, if a user exhibits high day-to-day variability (due to a variety of reasons), more data points will be needed for the algorithm to detect ovulation. If the available data is not sufficient for the algorithm to detect or exclude an increase of the BBT, the cycle is excluded from the analysis.

Please see our revision to the text underlined in the response to the previous comment.

Original comments: Ref (line 239) is from 2007 there should be more recent ref used to support the use of 24 days.

Original response: Line 239. The definition of normal cycles often used in research is 21-35 days long, please could the authors explain why they chose 24 days as the shortest cycle to include? • We used criteria from FIGO ([https://pubmed.ncbi.nlm.nih.gov/17362717/](https://urldefense.com/v3/__https:/pubmed.ncbi.nlm.nih.gov/17362717/__;!!LIr3w8kk_Xxm!-_bfMTl4-Gu0gC0t2RJ6KbytuyVi-qFyn179-vykm_0zm7tjUKe8VXoF2o9wKi4$)), which are based on more recent population estimates at the 5th and 95th percentile. Had we used 21 days, we would have significantly underestimated frequent menstruations and potentially been less likely to find a difference.

Comment 2: The reference the authors have provided is dated 2007, almost 14 years ago. There are many updated references that should be provided for clarification of their use of 24 days as the shortest cycle length to include. Please update.

We have updated the manuscript to include a reference from Munro, published in 2018: “The two FIGO systems for normal and abnormal uterine bleeding symptoms and classification of causes of abnormal uterine bleeding in the reproductive years: 2018 revisions.”
<https://pubmed.ncbi.nlm.nih.gov/30198563/>

Comment 2: Figure 1 is still very poor. I would suggest removing the background lines. Ensure all text is black font to align with the manuscript.

We have removed the background lines and noted that the text was previously a shade of grey, which we have now revised to black to align w/ the manuscript.

Original comment: Lines 299-301 The authors state a significant change in cycle length yet looking at the numbers this changes by 0.5 day, the authors need to clarify the length of cycles users inputted as it seems somewhat strange that a user would have a cycle length of 29.5 days.

Original Response: The cycle lengths are averaged from the number of cycles that are contributed over the study periods, which is why the averages are fractions of whole numbers.

Comment 2: My comment has still not been fully addressed here I apologise if I was not clear originally. My query is regarding the reporting of the cycle through the app. Generally, participants would report their cycle lengths in whole days. Is it feasible for a participants/users cycle to be reported for example as 29.5 days if they reported precisely in the day when bleeding started/ stopped and began again for the next cycle? Is this something that is feasible through the app? I fully appreciate and understand that the 0.5 increase would be an average.

We believe that the reviewer is asking if the app has the resolution to allow users to log half days in the app or utilize the timing of data entry to consider half days. However, we only account for full days, which is more appropriate given that timing of data entry may not necessarily match up with the event occurrence.

Original comment: Discussion: As per above there needs to be more a more detailed discussion of the possible limitations which should include the ovulation reliability, possibility of peri menopause, as well as the possible bias to the data set as already included. I would suggest a specific limitation section should be included as the whole basis of the data collection is reliant upon people taking their own ovulation testing and truthfully inputting the data. There are many studies that have discussed the inaccuracies of self-report data but none have been acknowledged or references. Please include such.

Original response: We note that the reviewer is very focused on a biased critique of the app being unable to accurately predict ovulation. We note that ovulation in all the possible ways in which it can be measured non-invasively are soft markers. Even the gold standard of using ultrasound cannot determine if a ruptured follicle has released an egg. To suggest that our research would be improved via a study that demanded transvaginal ultrasound and routine LH kit testing would be infeasible at our sample size. Ovulation prediction is limited, which is why our investigation additionally includes other menstrual parameters aimed at increasing our ability to detect changes related to the pandemic. The reviewer’s comments do not acknowledge that this data set is one of largest to collect these data and that any data collection performed in a clinical setting would be subject to even greater limitations. Any paper-collected self-report would be even less reliable and more likely to reflect recall bias, which our methods avoid by requiring daily data entry. Abiding by the reviewer’s request would turn a strength of our methodology into a weakness.

Original response: That being said, we have added the following broad limitation acknowledgement in a separate limitations section as follows: “The findings from this study are broadly limited by self-reported data. However, we believe that these data are collected from a group of individuals who are using the Natural Cycles app for its intended purpose of pregnancy prevention, rather than for the purposes of this research, such that these data are expected to be reliable.”

Comment 2: I have already acknowledged the large data set in the very first comment to the authors. Nor have I ever suggested an alternative more invasive method be used such as transvaginal US. I have focused on the ovulation due to the previous lack of detail provided, therefore making it difficult to understand as a reader how an app would predict ovulation. The information that has been added has certainly now helped the manuscript. However, it is still important to acknowledge the limitations, as with all original research. The reporting of basal body temperature, despite being daily, is still reliant on self-report as it is trusting participants will not falsify any data, as is the same with all self-report data. It does not mean to say your data is not reliable it is just something that cannot be guaranteed. Therefore, I would suggest adding to your acknowledgment of this with a reference regarding the reliability of self-report data.

Thank you for the clarification. Our discussion section includes a statement saying that the findings are broadly limited by self-reported data. However, we have additionally included references discussing the acceptability of self-reporting of menstrual data and proof of use and reliability from a previous publication. We also suggest that future studies may be improved by the use of wearable devices that can provide real-time data collection and transmission:

“The findings from this study are broadly limited by self-reported data. However, we believe that these data are collected from a group of individuals who are using the Natural Cycles app for its intended purpose of pregnancy prevention, rather than for the purposes of this research, such that these data are expected to be reliable. Mobile app-based reporting of menstrual data is preferred to conventional paper diary recording methods [27]. Further, previous publications on the menstrual cycle characteristics of more than 120,000 women using the Natural Cycles app to prevent or plan a pregnancy noted their individual contribution of approximately 9 cycles of data [28], suggesting user compliance and acceptability of the method. Natural Cycles’ recording of daily menstrual and basal body temperature diaries thus provided us with robust data for exploring female reproductive health and physiology in the setting of widespread socioenvironmental change—the COVID-19 pandemic. Future studies may be improved by the incorporation of real-time body temperature data collected by wearable devices, such as the Oura ring [29].”

**Reviewer #3: This paper is significantly improved but still needs some edits to make it suitable for publication. Specific comments below.**

Abstract: Much better! Only comment is that I would reword the last sentence both here and in the Discussion to read "... their experiences of the COVID-19 pandemic might differ ..."

Revised as suggested.

Introduction: Significantly improved. You still need to end this section with a clearer sentence on the objective of the study. Most papers end with a line that literally states "The objective of this study is to ..."

We have revised the introduction to be more clear about our primary and secondary objectives as follows:

“We conducted analyses using aggregated, real-world menstrual cycle data from app users both before and during the COVID-19 pandemic with the primary objective of detecting potential changes in ovulation, cycle length, and menstrual duration during the pandemic. The secondary objective of this study was to examine the association of any menstrual changes with self-reported perceived stress related to the pandemic.”

Methods:

-Page 6, Lines 123-125: This seems unnecessary and like an ad for the app.

We have removed the lines on the app’s contraceptive efficacy.

-Page 6, Inclusion/Exclusion Criteria: Were cycles of users who became pregnant during use included?

In the raw dataset we excluded cycles where the user was pregnant as well as the cycle following the end of the pregnancy. The manuscript has been updated to reflect exclusion of any cycle data during a pregnancy.

-Page 8, Lines 171-172: This was stated earlier and is redundant.

It’s unclear if the reviewer’s line numbers used the tracked version versus the clean version. If referring to the clean version and commenting on our clarification about excluding cycles with low data quality, we did so to address ambiguities raised by Reviewer 1. If the reviewer was referring to the tracked version where we talk about our data collection going from March through September, the repetition adds further clarity on how cycles were included if they spanned more than 1 month. For example, a cycle starting in September might not end until October but we clarify in our statement that it would be included. Without this statement, a reader might assume that we would only include cycles that had to start in and end in September. Doing so would then underreport long cycles.

Results:

-Relationship status in Table 1 seems to need a +.

Thank you for noticing this detail. We have added the footnote.

-It is unusual to present 95% CI for means, instead of SD. Please present that in the Methods and address why.

While it may be unusual, we believe that the 95% CI is easier for the lay reader to interpret rather than the SD, which might require the reader to take into account sample size.

-I continue not to think that both Figure 1 and Table 3 are both necessary, especially as there are no differences and this can be just addressed in the text.

We would defer to the editors of PLOS ONE to determine the value of the figures and tables, taking into context their publishing limitations.

-Page 13, Lines 243-247: Creation of the model should be in the Methods section. However, I do not see that you actually created a model. Table 4 is just a list of significant associated risk factors and is not a model.

We removed any mention of modeling and specified that we conducted a multinomial logistic regression.

Discussion:

-My biggest concern is that you take 1.5 pages of a 2.5 page discussion on a theory that defends your lack of expected results. It is ok to mention this but not to take up this much space on it. Instead, spend some time discussing the implications of your actual results.
Page 16, Lines 284-289: I don't understand how this is relevant.

We have streamlined the paragraph significantly and removed the extensive review of details from the references, which we agree do not add significantly more to the discussion.

-Page 17, Lines 312-315: Too much rehashing. Would rewrite as "Only 56.9% of users .... bias; however, this sub-analysis ..."

Revised as suggested.

-Page 18, Limitations: You could consider the possibility that the chosen time points were not enough to capture changes in menses and ovulation, as it might take more time to see the physiologic effects on stress, as COVID-19 did not start until mid-March 2020 and most thought it would be very temporary at that time.

Thank you for this point which we have included in the manuscript as follows:

“For this population, general stress provoked by the COVID-19 pandemic and its preoccupations may not have been sufficient to elicit an adrenal and adaptive neuroendocrine response to acute stress or chronic stress. We note as well that our chosen time points may not have been sufficiently long enough to observe stress-related physiologic effects which might not have been observed during the early months of the pandemic. Alternatively, our surface-level measurement of self-reported stress may have been insensitive to true distress or the behavioral determinants of stress-induced hypothalamic dysfunction [25]”

Conclusion:

-Page 19, Line 350: Per my comment in the Abstract, would change this to "may"

Changed as suggested.

-Suggest ending with a line "Future research is needed to ..."

We added a line about the importance of future studies that use wearable devices that better track data by avoiding self-reporting issues.
