# Supplementary material for: Detecting variations in ovulation and menstruation during the COVID-19 pandemic, using real-world mobile app data
Source: PLoS One. 2021 Oct 20;16(10):e0258314. doi: 10.1371/journal.pone.0258314 (PMC8528316; doi:10.1371/journal.pone.0258314)
Supplement: Attachment — Submitted filename: COVID Cycles 2021_09_21 - PLOSONE Review Response.docx [file pone.0258314.s003.docx]

**PLOS ONE Review Response #3**

Dear editors and reviewers,

Thank you for your favorable review of our manuscript, “Detecting variations in ovulation and menstruation during the COVID-19 pandemic, using real-world mobile app data.” We acknowledge the reviewers’ comments and have made revisions as appropriate with responses below. Please find our review responses highlighted in yellow.

Reviewer #1: Thank you for addressing my points. The only remaining point is regarding the statement 'many missing data points' when discussing the data that is included for final analysis. Please could the authors change this precise statement to make more quantifiable and perhaps in relation to the '10 temperature readings' that are stated to have been required earlier. This is my only remaining query.

Thank you for your query about the ambiguity in our manuscript. However, the explanation of how cycles are excluded is not as simple to explain as to provide a cutoff or threshold value. The quantity of missing data that is acceptable is a function as well of the stability of the user’s data as well. To help the reader understand this and reduce the ambiguity in the manuscript we have revised as follows (we have removed mention of “many missing data points”:

“If no temperature rise is observed and the data quality and quantity is deemed sufficient, the cycle is flagged as anovulatory. Of note, users with stable measurements (e.g., small day-to-day variations in the same cycle phase), require fewer data points for the Natural Cycles algorithm to draw conclusions about changes in the basal body temperature. Cycles with low data quality (e.g., high day-to-day temperature variability) or data that is insufficient to detect or exclude an increase in the basal body temperature are excluded by the analysis.”

Reviewer #3: Thank you for your thoughtful and substantial edits. The paper is now in very good shape! Only 2 comments:

1) Would recommend slight edits to the Limitations section. This seems to be a continuation of the paragraph above, rather than a clear statement of the limitations of the study, which does not clearly begin until >1 paragraph later. You could even just remove the Limitations subheading, and it would read better.

- Thank you for your comment, for which we’ve removed the “Limitations” subheading to help with the flow of the discussion section.

2) I continue to note that I do not think that both Figure 1 and Table 3 are both necessary, especially as there are no differences, and this can be just addressed in the text. However, this should not stop the paper from being published in its current form.

- Per editor, Fig 1 and Table 3 may remain in final text.
